# It’s the power of food: individual differences in food cue responsiveness and snacking in everyday life

**DOI:** 10.1186/s12966-015-0312-3

**Published:** 2015-12-07

**Authors:** Benjamin Schüz, Natalie Schüz, Stuart G. Ferguson

**Affiliations:** Division of Psychology, School of Medicine, University of Tasmania, Private Bag 30, Hobart, TAS 7001 Australia; School of Health Science, University of Tasmania, Hobart, Australia; Divisions of Medicine and Pharmacy, School of Medicine, University of Tasmania, Hobart, Australia

**Keywords:** Power of Food, Cue reactivity, Stimulus control, Snacking, Discretionary food choices, Ecological momentary assessment, Ambulatory assessment

## Abstract

**Background:**

Discretionary eating behaviour (“snacking”) is dependent on internal and external cues. Individual differences in the effects of these cues suggest that some people are more or less likely to snack in certain situations than others. Previous research is limited to laboratory-based experiments or survey-based food recall. This study for the first time examines everyday snacking using real-time assessment, and examines whether individual differences in cue effects on snacking can be explained by the Power of Food scale (PFS).

**Methods:**

Ecological Momentary Assessment (EMA) study with 53 non-clinical participants over an average of 10 days. Multiple daily assessments: Participants reported every snack and responded to randomly timed surveys during the day. Internal and external cues were measured during both types of assessment. Demographic data and PFS scores were assessed during a baseline lab visit. Data were analysed using multilevel linear and multilevel logistic regression with random intercepts and random slopes as well as cross-level interactions with PFS scores.

**Results:**

Higher individual PFS scores were associated with more daily snacking on average (*B* = 0.05, 95 % CI = 0.02,0.08, *p* < .001). More average daily snacking was associated with higher BMI (*B* = 1.42, 95 % CI = 0.19,2.65, *p* = .02). Cue effects (negative affect, arousal, activities, company) on snacking were significantly moderated by PFS: People with higher PFS were more likely to snack when experiencing negative affect, high arousal, engaging in activities, and being alone compared to people with lower PFS scores.

**Conclusions:**

PFS scores moderate the effects of snacking cues on everyday discretionary food choices. This puts people with higher PFS at higher risk for potentially unhealthy and obesogenic eating behaviour.

## Background

The notion that much of our eating behaviour is not guided by a physiological need, but by the response to food-related cues [1, 2] is well documented. Individuals are exposed to a multitude of food–related cues in their everyday lives, and both the number and density of these food-related cues have been implicated in whether someone eats or not [2–5]. In particular discretionary food choices (“snacks”), often defined as food consumed outside of main meal occasions [6], seem to be more influenced by such cues than main meals [7]. The factors that influence snacking are of great interest, as snacking seems to be linked to higher overall caloric intake and, accordingly, the development and maintenance of obesity and obesity-related health problems [8, 9]. Here we explore how people respond to internal and external snacking cues, and, importantly, whether there are individual differences in how these cues relate to snacking behaviour that can be explained using psychological variables.

### Environmental cues and snacking

Previous studies suggest that the majority of our eating—and our snacking in particular—is elicited by internal and external cues [1, 2, 10, 11]. For example, a longitudinal study [7] found that internal and external cues, rather than hunger, were the most frequently recalled triggers of snacking behaviour. These cues are mostly internal states (e.g., affect, arousal or stress), situations, or objects that have previously been associated with food intake [4]. Examples include seeing or smelling food, observing people eating, advertisements (external cues), being stressed, or mood states and desires for rewarding experiences (internal cues). It has been argued that exposure to these cues triggers the expectation of rewards through food, which might be misinterpreted as hunger [12], prompting eating. In addition, by observing other people eating, an implicit eating norm could be created which then makes food choices or the amount consumed more or less acceptable [13]. Current affective states can function as cues as well: for example, daily stressors [14] and social exclusion [15] have been linked to increased snacking, potentially because snacking can serve the function to improve negative mood [16]. Similarly, using data from the present study, we have recently shown that everyday eating and drinking behaviour can be predicted by such internal and external cues [5]. Research into these situational determinants of eating is paramount, because it furthers our understanding of eating and because it can suggest avenues for intervention to change obesity-related eating behaviours.

However, not everyone responds to internal and external food-related cues the same way. Some cues appear to be more relevant to some people, or put another way, some people might be more sensitive to cues in general and accordingly consume more food [17, 18]. One psychological mechanism that might be responsible for individual differences in snacking following food-related cues are individual differences in the degree to which food-related cues affect eating behaviour [1]. This logic underpinned the development of the Power of Food Scale (PFS) [19, 20], which was designed to measures individual differences in being aware of food availability, reactions to thinking about food, and reactions to tasting food. Available evidence suggests that the PFS is particularly relevant for predicting snacking behaviour. For example, a recent large-scale study [21] showed that higher levels of the PFS predicted more snacking. Thus, this scale has great potential to be a useful screening tool to identify people at risk for potentially unhealthy food choices in response to particular cues – but this link is so far missing, at least for everyday snacking.

Work conducted to date that examines the validity of the PFS in predicting food choices has either been conducted in tightly controlled laboratory settings [22, 23], or been conducted retrospectively using food frequency questionnaires [24], clinical interviews [19] or other forms of dietary recall. These forms of research may not depict eating behaviour in the context where it happens— that is, everyday life. As such, it is yet to be examined whether the PFS actually measures responsiveness to cues, limiting both the clinical utility of this scale and our underlying understanding of the factors that influencing snaking behaviour.

### Aims of the present study

Both the type and frequency of snacking events have been implicated in hypercaloric diets [8], and it has been shown that discretionary food choices are responsible for up to a third of our total dietary energy intake [24]. Identifying potentially modifiable factors that influence snacking is paramount for preventing and treating obesity. Previous studies have underlined the importance of internal and external cues for snacking behaviour [3, 5, 7, 11, 13, 25]. In this study, we aim at providing further evidence for the validity of the PFS in explaining day-to-day snacking behaviour by examining whether the effects of environmental cues on snacking are moderated by PFS scores. Importantly, rather than relying on retrospective measures of triggers and dietary behaviours, we use ecological momentary assessment (EMA) [26] methods to collect real-time data on eating and environmental stimuli. This technique overcomes many of the limitations in recall-based eating assessments such as biased memory and memory lapses [27], and in addition is able to capture the presence or absence of food cues present during everyday eating.

## Methods

As part of this EMA [26] study, participants were asked to carry a custom-programmed mobile phone with them throughout their everyday life and log every episode of eating and drinking as well as respond to random prompts, randomly timed assessments. Internal and external cues were assessed both at eating and randomly timed assessments; this allows comparing the presence and intensity of these cues between eating logs and random prompts [25]. In a previous publication based on this data set, we have shown that everyday dietary behaviours are strongly associated with internal and external cues [5]. In this study, we examine whether the importance of these cues for snacking varies between individuals and whether the PFS scale can explain these differences.

### Participants and procedure

For this study, 53 participants (41.51 % female) aged 18–60 years (*M* = 28.17 years, *SD* = 11.15) with a BMI range between 17.7 and 37 (*M* = 23.9, *SD* = 4.14) were recruited via community advertisements and through ads on the Facebook® social media website [28] asking for individuals interested in participating in a study examining “eating patterns”. Inclusion criteria were being over 18 years of age, having no history of an eating disorder, and not currently trying to change or restrict eating patterns (e.g., being on a diet). Ethical approval was obtained from the Tasmanian Social Sciences Human Research Committee (H.0012474).

Detailed procedures have been published elsewhere [5], and the monitoring protocol was similar to previous research in the area [25, 28–30]. Briefly, during an initial visit to the research laboratory and after providing informed consent, participants were measured and weighed (for BMI computation) and were asked to fill in a baseline questionnaire assessing sociodemographic information as well as the Power of Food Scale [19, 20]. This scale assesses individual differences in the psychological influence of the food environment using 15 items, e.g., “I find myself thinking about food even when I’m not physically hungry” to be answered on a five-point Likert scale from 1 “I don’t agree” to 5 “I strongly agree”. PFS scores can accordingly vary between 15 and 75.

Participants then completed ten days of field monitoring, with a brief visit at the study centre to assess protocol compliance and troubleshoot potential technical issues. During field monitoring, participants were asked to carry the EMA device during waking hours and log (by pressing a button) every instance of eating and drinking. When logging eating behaviour, participants could choose between reporting a meal (breakfast, lunch, or dinner), or a snack, which was defined as any item of food eaten between the main meals of breakfast, lunch, or dinner [31]. Participants were instructed to log events immediately before consumption. In addition to logging their eating and drinking, participants were required to respond to 3–5 randomly timed prompts during the day. Each reported event was date- and time-stamped. In addition, participants completed a daily evening report in which they had the opportunity to report any snacks they forgot to log during the day.

During each log of food or drink consumed and for each random prompt, participants were asked to indicate their affect and arousal state (internal cues) *at the time they decided to eat* using 14 adjectives derived from the circumplex model of affect [32]. Individual items were presented on-screen, one item at a time and answered using a slider (e.g., “Happy?” with a visual analogue slider scale ranging from “No!!” [0] to “Yes!!” [100]). The items were adapted from previous EMA studies on smoking [33, 34]. Responses to the individual items were then converted to z-scores (within-subject; *M* = 0, *SD* = 1.00), and a maximum likelihood factor analysis (using geomin rotation) controlling for non-independence of observations using the TYPE = COMPLEX option in Mplus was used to extract two factors based on eigenvalues > 1 (affect, Cronbach’s alpha = .82 and arousal, Cronbach’s alpha = .71), with higher scores indicating more negative affect or higher arousal, respectively.

Participants were further asked to indicate external cues in four domains—social setting (e.g., whether they were with partner or with friends), activities (e.g., working, engaging in leisure activities (generic), or being between activities), social cues (seeing someone else eat), and the availability of different foodstuffs (e.g., savoury foods, confectionary). The assessment of the first four domains were based on context factors identified in smoking-related EMA work [33, 35], the assessment of the availability of foodstuffs was based on previous EMA work on eating [25].

### Analyses

Because EMA data has a hierarchical structure in which the repeated daily assessments of snacks are nested within an individual, we used multilevel regression analyses with random intercepts, random slopes and cross-level interactions of the PFS with intercepts and slopes [36] to account for this structure. We then examined for each external and internal cue separately whether cross-level interactions existed between the intercepts and the slopes on level-1 (measurement occasion) and PFS scores on level-2 (person). For these analyses, all level-1 predictors were group-mean centred, and Power of Food on level-2 was grand-mean centred, following current recommendations [37]. The availability of food was coded as 1 (food present), 0 (no food present), social setting was coded as 1 (other people present), 0 (no other people present), activities were coded as 1 (engaging in activities), 0 (being between activities), and social cues to eating were coded as 1 (seeing others eat) or 0 (not seeing others eat). The factor scores for affect and arousal were computed as described above.

## Results

A detailed summary of the data collected during monitoring has been reported elsewhere [5]. Overall, during the monitoring period, a total of 1056 snack reports (*M* = 1.75 snacks per participant day) were completed, and of 2057 random prompts issued, 1870 (90.1 %; *M* = 3.11 per participant day) were answered. Another 250 snacks were reported retrospectively in the evening reports (these reports were not subjected to a full assessment). Participants consumed an average (mean of means) of 2.17 snacks per day.

PFS scores (*M* = 40.00, *SD* = 12.18) correlated moderately positively with BMI (*r* = .28, *p* = .04). Females indicated higher PFS scores, but these sex differences were small (*r* = .10) and not significant (*t*(54) = 0.82, *p* = .42).

### PFS, snacking, and BMI

**Fig. 1:**
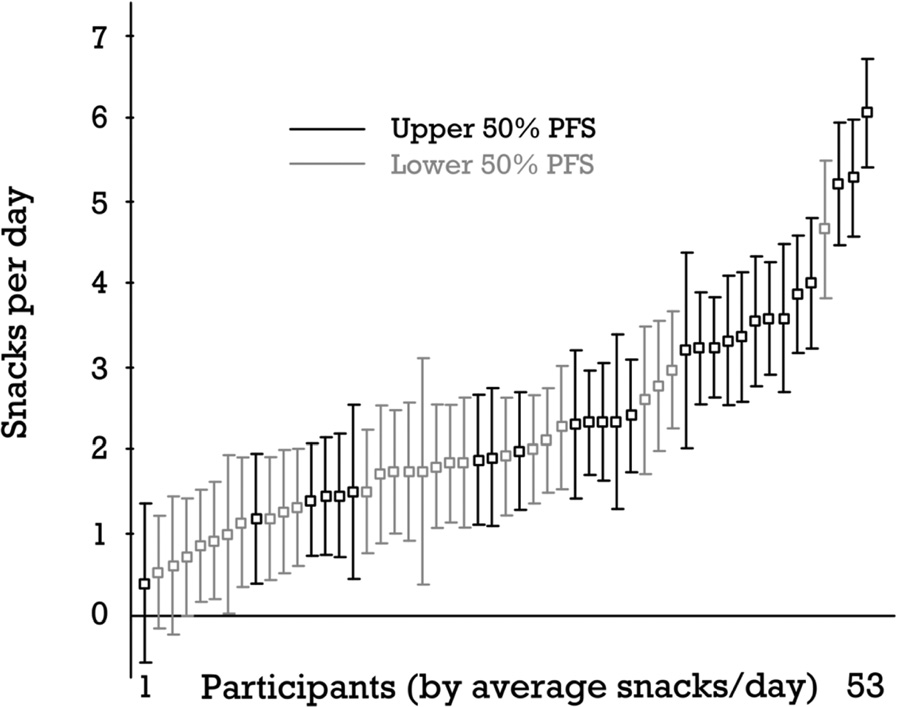
Participants ordered by average number of snacks per day and pfs scores. Note. 95 % confidence interval around the average number of snacks per day

The intraclass correlation coefficient (ICC) of the number of snacks per day was *ρ* = .46, indicating that almost half of the variation in the number of snacks was due to differences between participants. To examine whether PFS affected the overall number of snacks per participant and day, we predicted the individual means of snacks per day (obtained in snack reports and evening reports) from individual PFS scores in a multilevel regression analysis, and we found that participants’ PFS scores (level-2 variable) significantly predicted the intercepts of snacks per day (*B* = 0.05, *SE* = 0.01; *p* < .001). This means that someone with a PFS score 20 points higher than another person(range of the PFS: 15–75) will consume one additional snack per day on average compared to the latter person. Figure 1 illustrates this and shows the individual means of snacks per day sorted by median snacks per day and PFS score.

To examine whether the daily frequency of snacking was related to individuals’ BMI, we regressed participants’ BMI on the random intercepts of the mean frequency of daily snacks in a subsequent multilevel model. We found that the average daily number of snacks significantly predicted BMI (*B* = 1.42, *SE* = .63, *p* = .02): a difference in the daily average snack number of 1 snack was associated with an increase in BMI of 1.42 points.

### PFS and the effects of internal and external cues

The ICC of snack reports vs. random prompts was ρ = .10, which indicates that a substantial part of the overall variation in the likelihood of snack reports is due to differences between participants [36].

**Fig. 2:**
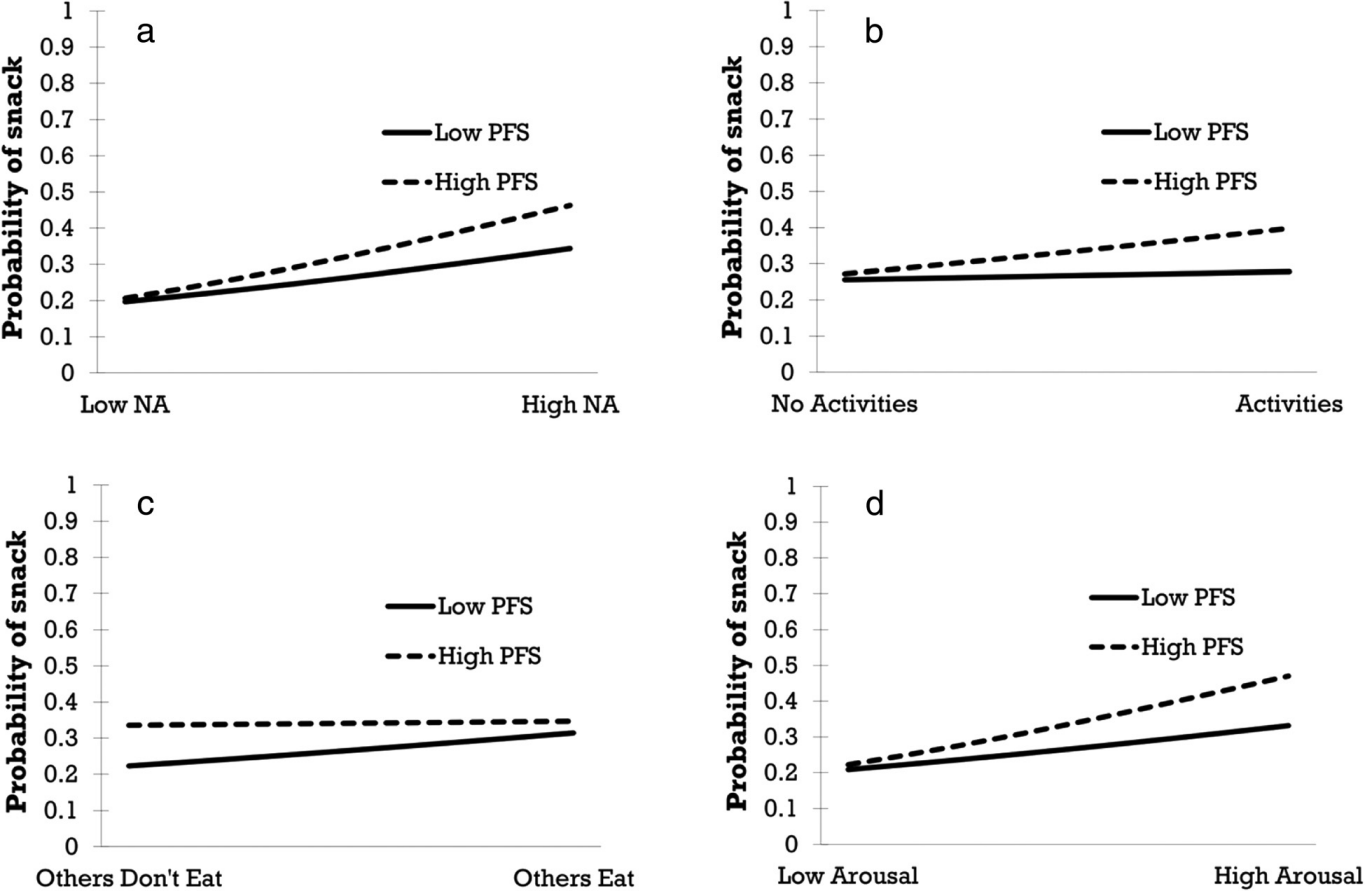
Interactions between cues (level-1, measurement occasion) and power of food (level-2, person) in predicting the probability of a snack report

To examine the effects of internal and external cues and the PFS on snacking, we compared snacking reports and random prompts with regard to the presence of external cues and the intensity of the internal cues. For each assessment (random prompt and snack report), we predicted the likelihood that this specific report is a snack report or a random prompt from the presence (categorical cues) or intensity (affect and arousal) of the external and internal cues in a multilevel logistic regression analysis. These analyses fit and pool the results of individual logistic regression models for each participant, in which the intercepts (baseline likelihood of snacking) and the effects (slopes) of the cues on snacking are allowed to differ between individuals, and we then predicted differences in intercepts and slopes between individuals from individual differences in PFS scores (Table 1). Significant interactions between the intercepts and PFS indicate that individual PFS score can predict individual differences in the average probability of snacking compared to random prompts, whereas significant interactions between the PFS and the slopes indicate that PFS scores can predict differences in the effects of the cues on snacking. Significant interactions were probed at −1 SD and + 1 SD of the moderator PFS [38] and the likelihood of eating snacks was plotted for these interactions against the likelihood of a random prompt (Fig. 2). We found that individuals higher in PFS are more likely to consume snacks when experiencing negative affect (Fig. 2a) or engaging in activities (Fig. 2b) compared to individuals low in PFS. The figure indicates that for example the likelihood of a snack compared to a random prompt increases to around 0.48 for someone high in PFS when experiencing negative affect as opposed to around 0.34 for someone low in PFS. Further, observing others eating seems to mainly increase the snacking behaviour of individuals low in PFS (Fig. 2c) up to the level of individuals high in PFS. With regard to arousal, individuals higher in PFS were more likely to snack when in higher arousal states than individuals lower in PFS (Fig. 2d).

**Table 1:** Odds Ratios of Cues (Within-Participants), Power of Food Scale Scores (Between-Participants) and Interactions Predicting Snacking (Reference Category = Random Prompts)

		Odds ratios (95 % CI) of internal and external cues (Covariates)					
		Negative affect	Arousal	Activities	Food available	Others eat	Company
Fixed effects	Intercept	0.25 (0.22,0.28)***	0.25 (0.22,0.28)***	0.24 (0.21,0.28)***	0.22 (0.19,0.25)***	0.23 (0.20,0.27)***	0.24 (0.22,0.27)***
	PFS*Intercept	1.01 (1.00,1.02)***	1.01 (1.00,1.02)**	1.01 (1.00,1.02)**	1.02 (1.01,1.03)**	1.02 (1.01,1.03)**	1.01 (1.00,1.02)*
	Slope Cue	1.11 (0.94,1.31)	1.03 (0.87,1.22)	0.52 (0.36,0.76)***	5.23 (3.45,7.95)***	4.06 (3.21,5.13)***	0.66 (0.50,0.88)**
	PFS*Slope Cue	1.01 (1.00,1.02)*	1.01 (1.00,1.02)*	1.03 (1.00,1.06)*	0.99 (0.96,1.02)	0.97 (0.96,0.99)**	0.98 (0.97,1.00)
Random effects (Residual variances)	Intercept	0.08**	0.08**	0.07*	0.11**	0.10**	0.08**
	Slope Cue	0.002	0.04	0.72**	0.63*	0.02	0.17

Note. *p < .05, **p < .01, ***p < .001

## Discussion

This study for the first time examined whether individual differences in appetitive behaviour towards food as measured by the Power of Food Scale (PFS) [19] manifest in differences in day-to-day snacking and the real-time effects of internal and external food cues in everyday life. In 53 non-clinical individuals, we found that higher PFS scores predicted a higher average number of snacks per day. Further, we found that the degree to which internal (negative affect) and external (availability of foodstuffs, social cues, activities) cues affected snacking (defined as discretionary meals apart from the main meals), was moderated by individual PFS scores. This is of particular importance, since a higher number of snacks per day was also related to a higher BMI, and because consuming more energy-dense discretionary foods has been associated with higher obesity prevalence and more negative health outcomes [8, 9].

### Power of food and snacking

Our study is the first to show that individual PFS scores predict the probability of everyday snacking, with higher PFS scores predicting more snacking. As demonstrated, consuming more snacks per day is associated with higher participant BMI. In previous research, consuming more energy-dense discretionary foods has also been associated with a higher risk for obesity and other health problems [8, 9], which puts people with higher PFS scores at higher health risk. This finding replicates previous studies that have shown that people scoring higher on the PFS report a higher frequency of consuming energy-dense snacks [21], but goes beyond previous work in demonstrating these effects in everyday snacking and in close to real time. Further, previous studies relied on food recalls, which are subject to a range of potential limitations including memory biases [27]. The use of EMA in this study is a significant advance over this methodology, as it significantly reduces the chance for biased memory and further allows the examination of behaviour in close to real-time [5, 26].

### Power of food moderates the effects of eating cues

Apart from a main effect of PFS on the average number of snacks per day, this study also identified significant interactions between PFS scores and the effects of internal and external cues on the likelihood of snacking. These findings are of particular relevance, since they indicate that people with higher PFS might be at higher risk for discretionary food choices in specific situations.

Individuals with higher levels of PFS were more likely to consume snacks when they experienced higher levels of negative affect as compared to people with lower PFS scores. This is in principle in line with the idea of ‘comfort eating’ [16], which suggests that people might consume energy-dense foods in order to self-regulate negative mood. Our study suggests that this motive for snacking might be most prevalent in people with high PFS levels. This provides further evidence to the notion that the relation between mood and snacking might not be as straightforward, but be subject to moderators such as self-regulatory capacities [39] or indeed PFS scores.

There were also significant interactions between PFS and the effects of engaging in activities, with participants high in PFS reporting more snacking while engaging in activities than participants low in PFS. Due to model complexity we could not disentangle which activities participants engaged in, which means that it might well be that participants high in PFS engaged in other activities while snacking than participants low in PFS.

A different interaction effect was observed between PFS and the cue of others eating: individuals high in PFS consumed more snacks than individuals low in PFS when no others ate, and this significant difference was attenuated by the presence of others or others eating. This might have to do with a ceiling effect in that these factors could not increase snacking any more in people high in PFS, or, more speculatively, with differences in social triggers to eat between low and high PFS participants. Other studies have reported differential effects of social factors [15] such that participants who habitually ate when socially excluded reduced eating when in company—a mechanism which could potentially underlie our findings as well. This effect is similar to observations from binge eating disorder - people with binge eating disorders eat more when alone [40], which also suggests differential effects of the social context on eating. Finally, participants higher in PFS consumed more snacks when in high-arousal states than individuals lower in PFS. Previous research has indicated that being aroused such as in stressful situations is a risk factor for snacking [14]; the results of our study add to this by suggesting that it might be in particular people high in PFS that are susceptible of responding to high-arousal states with snacking. Additional analyses (not reported here) that specified both PFS and BMI as simultaneous moderators of the cue-snacking association showed that for all cues examined, only PFS emerged as significant moderator, whereas BMI did not moderate these relationships. This further underlines the notion that psychological processes underlie the differential associations of cues and snacking according to PFS.

Interestingly, there are some similarities between the overall pattern of cues for those high in PFS and the cues identified in EMA research on smoking [41] and drinking alcohol [42], in particular with regard to the roles of availability and social cues (observing others performing the behaviour). This suggests at least some similarities in underlying pathways, in particular since it has been shown that PFS effects are not due to differences in state hunger in individuals [23], i.e., people with higher PFS scores do not experience more hunger, but react differentially to food-related cues. Instead, it has been suggested that individual differences in the PFS are reflective of underlying differences in the reward system [22], in particular in dopaminergic pathways that would then relate to differences in the sensitivity to food-related rewards [12].

### Strengths and limitations

A key strength is the fact that this study was the first to examine the role of PFS for everyday snacking and to responses to snacking cues in everyday life using EMA. EMA has significant advantages over diary- and other recall-based assessments, as it measures eating in close to real time [29]. Further, memory biases inherent in self-reports and recalls have been implicated in an under-reporting of snacks [43], which is prevented in EMA studies. Being able to compare the presence or absence of specific cues between eating logs and random prompts, and being able to assess this repeatedly within participants further allows examining the role of cues on snacking in more detail than in recall studies. However, the relative burden of EMA studies limits the reach and sample size; our study is limited by its relatively small sample of 53 adults. Previous simulation studies however indicate that sample sizes in this range are sufficient to detect the cross-level interactions that we were interested in [44]. In an effort to reduce participant burden, we chose to refrain from detailed snack assessments that would allow for estimates of caloric and nutritional content, which is a further major limitation of the study. This and the relatively small sample size precluded additional analyses, and so we cannot rule out and in fact suspect that the cues for healthy snacks (such as fruit) might differ from those triggering unhealthy snacks (such as chips). Future EMA research should examine differential triggers for healthy and unhealthy snacks, and more refined assessments of snacks that allow separating healthy from unhealthy snack foods. By limiting the assessments to ‘meals’ and ‘snacks’, we might have used other definitions of these eating behaviours than participants themselves might have used [6]. A further limitation is the fact that our data does not allow to control whether participants have accurately recorded all snacks consumed. However, through intensive one-on-one training following validated protocols [45], we have ensured that all participants were familiar with the study protocol and confident in using the EMA device correctly, thus limiting this potential bias. Further, the fact that our sample was predominantly white and well-educated prevents generalisation to the broader community. Ideally, the link between PFS and BMI via snacking would be established through formal mediation tests, and future studies with multiple assessments of BMI over time could provide evidence for such a causal pathway. Lastly, the selection of cues assessed in our study is only a limited selection of potential cues, and further research in the area should look at more cues. For example, we have not assessed known predictors of eating behaviour such as recurring daily hassles [14] or state hunger [23], and we have not assessed self-regulatory capacity or desire to eat at each measurement occasion, both of which have been implied in the consumption of energy-rich snacks and in the response to internal and external food cues [30, 39].

## Implications and conclusion

Together with previous studies on the role of PFS [19–21, 23], the present study has important implications for the prevention and treatment of obesity. Our study shows that these lab-based findings translate into everyday eating behaviours and put people with high PFS scores at higher risk for consuming discretionary foods and associated health problems. Our results also show that experiencing negative affect or high arousal or engaging in specific activities have stronger effects on consuming snack foods in people with higher PFS scores. In addition, we found that consuming more snacks per day was associated with higher BMI. This would suggest that behavioural interventions to prevent and treat obesity should take individual PFS status into account, since this might determine how individuals respond to internal and external food-related cues. Further, interventions need to take into account the cue dependency of eating snacks and target these cues in addition to aiming at increasing person-level factors such as self-control. Depending on individual PFS levels, specific cues might be particular risky in terms of triggering snacking behaviour, and would thus require specific attention in counteracting the effects of these cues.

## Abbreviations

BMI: body mass index (kg/(m^2^))
EMA: ecological momentary assessment
PFS: power of food scale
